# Competition in International Generic Drug Markets

**DOI:** 10.1001/jamahealthforum.2024.3391

**Published:** 2024-10-11

**Authors:** Étienne Gaudette, Shirin Rizzardo, Kevin R. Pothier, Mina Tadrous

**Affiliations:** 1 Institute of Health Policy, Management and Evaluation, Dalla Lana School of Public Health, University of Toronto, Toronto, Ontario, Canada; 2 National Prescription Drug Utilization Information System Research Initiative, Patented Medicine Prices Review Board, Government of Canada, Ottawa, Ontario, Canada; 3 Leslie Dan Faculty of Pharmacy, University of Toronto, Toronto, Ontario, Canada; 4 Women’s College Hospital, Toronto, Ontario, Canada

## Abstract

This cross-sectional study compares the small molecule generic drug markets of a group of high-income countries with similar pharmaceutical regulatory environments.

## Introduction

While expensive new medicines draw considerable policy attention, older and more affordable generic drugs play a pivotal role in global drug utilization. Having strong generic markets with multiple manufacturers has benefits for supply chain resiliency, drug plan sustainability, and, most critically, access to medicines.^1,2^

This repeated cross-sectional study compares the small molecule generic drug markets of a group of high-income countries with similar pharmaceutical regulatory environments. We compiled and contrasted metrics related to the competitiveness of generics in Australia, Belgium, Canada, France, Germany, Italy, Japan, the Netherlands, Norway, Spain, Sweden, the UK, and the US.

## Methods

We used sales data from the MIDAS database (IQVIA) to study each country’s generic market. Drugs were defined as unique arrangements of pharmaceutical ingredient(s) and strength(s), which allowed for products with different formulations to be considered potential competitors (eg, 100-mg tablets and 100-mg capsules of doxycycline). All prescription drugs were included in the analysis (ie, drugs of all formulations, provided by both retail and hospital sectors and with payers in all market segments).

For fiscal years (April-March) 2010 to 2022, we graphically presented the number of manufacturers selling 25 or more generic drugs in each country. While every drug has its own market, this indicator provides information about the overall competitiveness of generic drug markets and the potential for more suppliers to compete when patents expire. We then showed the proportion of off-patent products for which a single manufacturer accounted for more than 50% and 100% of sales. All analyses were performed using SAS, version 9.4 (SAS Institute).

## Results

**Figure 1.**
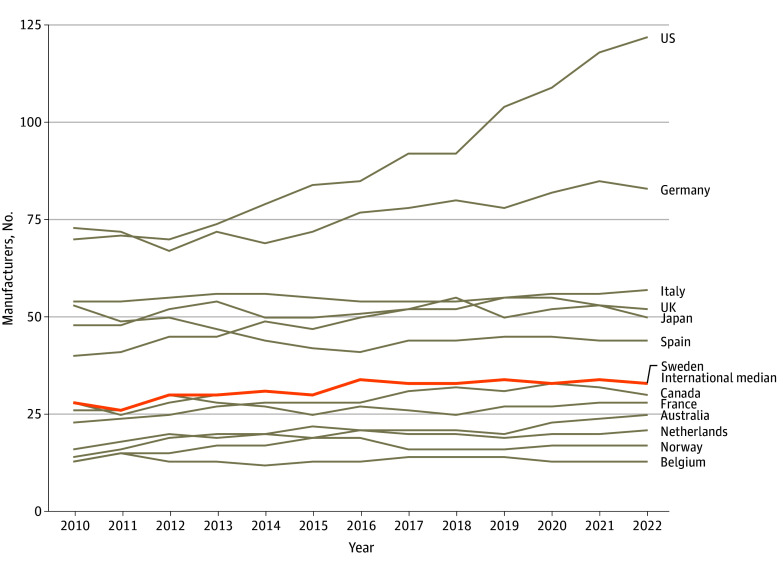
Generic Manufacturers Selling 25 or More Generic Drugs, 2010-2022 The threshold of 25 or more was chosen to count manufacturers with a considerable portfolio of drugs. Manufacturer names featuring suffixes and regional designations were truncated to avoid double counting (eg, Fresenius Medi.Jap was replaced with Fresenius). Generic company counts did not group subsidiaries and parent companies except for cases where the source data identified the subsidiary status in the company’s name.

Between 2010 and 2022, the number of generic manufacturers selling 25 or more generic products grew in 10 of 13 countries (Figure 1). Although levels were generally associated with population size, there were cases where this association did not hold. For example, Sweden had more generic manufacturers than France in 2022, despite having less than a sixth of its population.^3^ The US and Australia experienced the largest increases in relative terms, with 74% and 79%, respectively (US: 70 companies in 2010 and 122 companies in 2022; Australia: 14 companies in 2010 and 25 companies in 2022).

**Figure 2.**
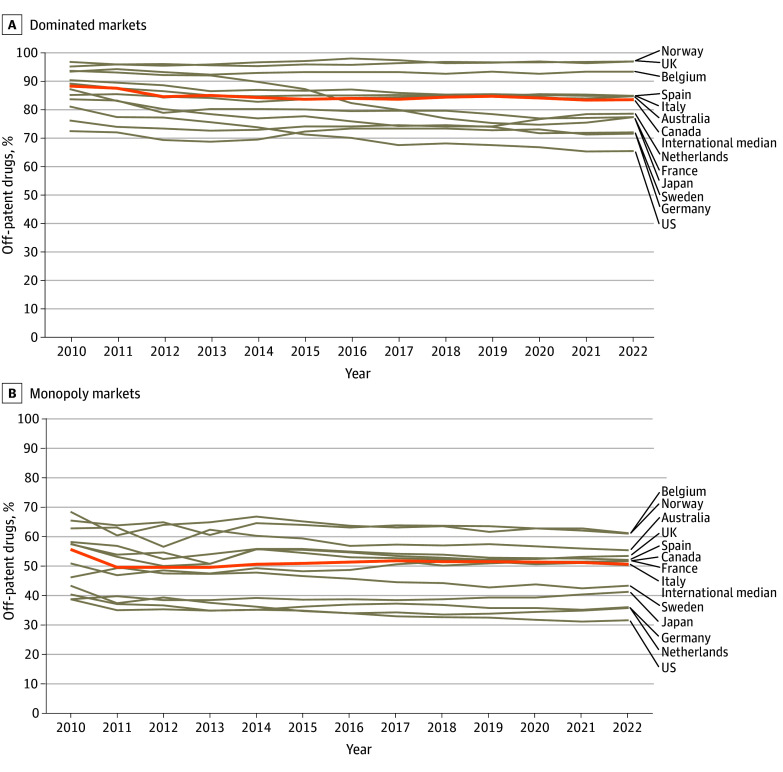
Proportion of Off-Patent Drugs in Dominated and Monopoly Markets, 2010-2022 The share of off-patent drugs for which the units sold (either flagged as generic or brand) by 1 company represented 50% or more (dominated markets) and 100% (monopoly markets) of total units sold. In each country, all off-patent drugs with sales were included in the calculations. A drug was considered off patent if at least 1 country recorded a sale for it categorized as generic in the source data.

The international share of off-patent drugs for which a company dominated sales was substantial during the study period. In 2022, all countries had more than 70% of off-patent products dominated by a single company, with the exception of the US (2260 of 3469 [65.1%]; Figure 2A). Furthermore, a majority of off-patent drugs were in a monopoly market in 8 of 13 countries (Figure 2B). Only 2 countries showed notable reductions in dominated and monopoly markets: Sweden (−15.1 percentage points [pp] and −12.3 pp, respectively) and the US (−15.5 pp and −11.8 pp, respectively).

## Discussion

In this repeated cross-sectional study, we found important international variations in 3 indicators associated with the competitiveness of generic markets. The US and Germany appeared most competitive based on the metrics analyzed, while smaller countries tended to seem less competitive. This is consistent with previous findings that market size is associated with generic competition.^4^

Despite more manufacturers recording generic sales in most countries since 2010, the present results had potentially concerning implications in the context of increasing drug shortages, a problem that has gained increasing attention over the past decade and is now considered a crisis.^5^ Market concentration remained intractably high in all countries in 2022, meaning that supply disruptions may cause patients to switch therapies or forego treatment for a large share of drugs sold in all countries. While drug shortages are complex phenomena and caused by multiple factors,^2^ policy efforts to strengthen competition (eg, incentivizing market entry of new manufacturers and deterring market dominance) could strengthen access to medicines.

This study has limitations. Patent protection timelines differ by country, such that drugs were counted as off patent while still under patent protection in some countries. Drugs that recently lost patent protection may not have reached market equilibria. Differences in metrics may be caused by the composition of drugs sold across countries in addition to true market differences.

## Notes

Supplement. Data Sharing Statement
