# Supplementary material for: Competition in International Generic Drug Markets
Source: JAMA Health Forum. 2024 Oct 11;5(10):e243391. doi: 10.1001/jamahealthforum.2024.3391 (PMC11470386; doi:10.1001/jamahealthforum.2024.3391)
Supplement: Supplement. — Data Sharing Statement [file jamahealthforum-e243391-s001.pdf]

Data Sharing Statement

Gaudette. Competition in International Generic Drug Markets. *JAMA Health Forum*. Published October 11, 2024. doi:10.1001/jamahealthforum.2024.3391

Data
Data available: No

Additional Information
Explanation for why data not available: The primary data are owned by IQVIA.
